# Evaluation of Ballasted Railway Track Response in Potentially Critical Areas Using Vibration Measurements

**DOI:** 10.3390/s25144363

**Published:** 2025-07-12

**Authors:** Mojmir Uranjek, Andrej Štrukelj

**Affiliations:** Faculty of Civil Engineering, Architecture and Transportation Engineering, University of Maribor, Smetanova 17, SI-2000 Maribor, Slovenia; andrej.strukelj@um.si

**Keywords:** ballast railway track, degradation, critical areas, welds, vibration parameters, measurements, mitigation measures

## Abstract

In railway infrastructure, particularly where concrete sleepers are employed, certain critical zones exhibit pronounced degradation of the ballast layer. Previous studies have identified several contributing factors, including the presence of welds, heterogeneity in the substructure beneath the sleepers, and variations in the track’s geometric parameters. Of these factors, the presence of welds seems to have the most significant influence. This article aims to determine whether differences in the ballast railway track’s response to traffic loads at weld locations can be identified in the initial phase, before obvious damage appears. Vibration responses in terms of displacement, velocity, and acceleration were measured on upgraded concrete sleepers equipped with rubber under-sleeper pads. The results indicate that velocities and accelerations at rail weld locations differ significantly from those in adjacent track sections, when the railway track is in an intact, undamaged condition. These results suggest a high likelihood of damage formation in these critical locations, indicating the necessity of preventive measures to mitigate damage. Possible mitigation measures that could help reduce the formation of damage are proposed.

## 1. Introduction

In Slovenia and other regions, particularly where concrete sleepers are employed, pronounced degradation of ballast railway tracks is observed in critical areas due to several contributing factors. These include the presence of welds (low or high quality, present on one or both rails), the condition of the layers beneath the sleepers (heterogeneity of layers, thickness of gravel), and geometrical parameters of the track (such as displacement, twist, and gauge). Among these, the presence of welds appears to be the most influential parameter [1]. There are also other important factors such as flat wheel impacts and others from vehicle-generated sources of dynamic loading, microstructural inhomogeneities of the rail and its surface, as well as irregularities in rail fastening systems [2]. In general, high rates of track degradation can be linked to singular features (rail bridges, level crossings, etc.), local geometry faults, sub-layers of inferior quality formation, and welds of inadequate quality [3]. Insulation joints can also represent a significant factor in ballast degradation, as they introduce stiffness discontinuities in the rail that disrupt uniform load distribution [4,5]. This article aims to determine whether the differences in response of ballast railway track to traffic load at the location of the welds could be identified in the initial phase, even before obvious damage appears. If the track’s response at the weld locations differs from that in the remaining areas, it suggests a high likelihood of future damage at the weld locations. Consequently, preventive measures to mitigate damage should be implemented in these critical locations in the initial phase, before damage occurs.

**Figure 1:**
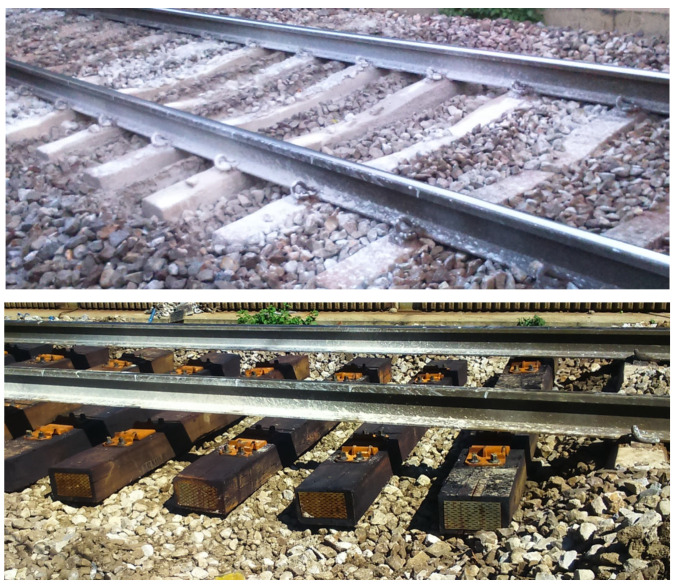
Replacement of worn concrete sleepers in a severely damaged area with new wooden ones.

In Slovenia, concrete sleepers are the main ones used in ballasted track construction; wooden sleepers are mostly used in areas of switches and crossings. One of the advantages of wooden sleepers is their ability to efficiently spread ballast contact forces over a larger contact area through ballast grain indentation, which concrete sleepers do not allow [6]. Therefore, in the case of concrete sleepers, the main issue arises at the contact interface with the ballast bed, where critical areas are subjected to uneven dynamic loading due to factors such as welds, subgrade heterogeneity, geometric irregularities, and other related influences. This results in localized stress concentrations, leading to ballast crushing and accelerated track degradation. Further advantages of wooden sleepers are simple manipulation and installation, greater elasticity, and consequently reduced noise during train operation [7]. Although wooden sleepers have many advantages and can, at least in critical areas, exceed the life span of concrete sleepers, they require more frequent maintenance, mainly due to the loosening of the fixing material. Further disadvantages of wooden sleepers include the demanding maintenance required to preserve track gauge, the risk of rotting over time, and the use of ecologically questionable substances for wood impregnation [8]. The current practice in Slovenia, in severely damaged areas experiencing pronounced degradation of the ballast railway track, typically involves replacing worn concrete sleepers with new wooden ones and installing a new ballast layer, as illustrated in Figure 1.

The results of the measurements prove that this replacement is effective, at least as a temporary solution, since wooden sleepers respond more favorably to traffic loads than concrete sleepers. However, the durability of wooden sleepers is generally problematic, so more appropriate and durable solutions are necessary in areas where damage is expected.

The problem of damage formation in critical areas of the ballast railway track is also being addressed by installing modified concrete sleepers with rubber under-sleeper pads (USPs). By using such sleepers, the contact area between the ballast bed and the sleeper’s lower surface is increased, which allows a reduction in contact stress and consequently contributes to the reduction of damage [9,10]. Similarly, as with wooden sleepers, ballast contact forces are spread over a larger contact area, although wooden sleepers still represent a cheaper alternative compared to the use of USPs on concrete sleepers [11]. The results of the measurements of the vibration parameters of the improved concrete sleepers with USPs, as presented in this article, confirm that, even in an undamaged state, the response at the location of the welds differs from that in areas without welds. Several studies have shown that, although USPs offer substantial benefits during the initial stages of track operation, their protective effects tend to diminish over time—especially in heavily loaded track sections or where the ballast layer is reduced [12,13]. Therefore, due to the high likelihood of damage formation, the implementation of additional preventive measures is recommended in such critical locations.

## 2. Materials and Methods

### 2.1. Setup of Vibration Measurements

**Figure 2:**
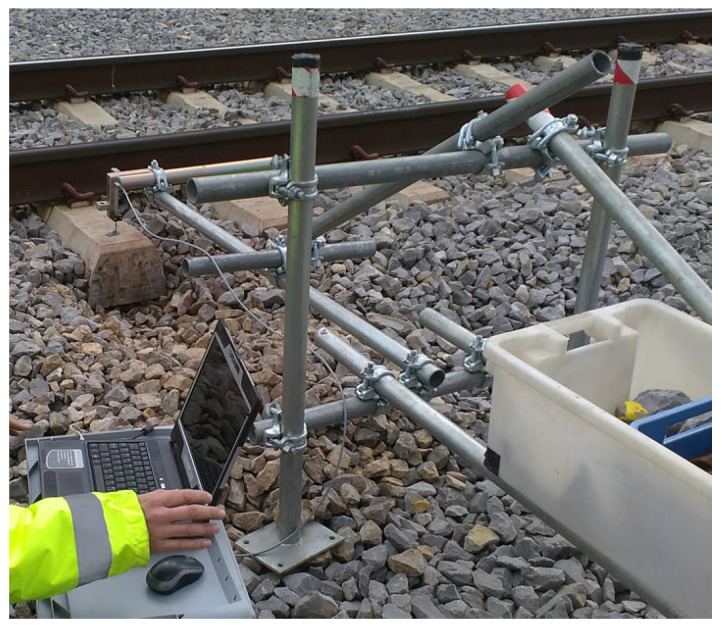
Cantilevered scaffolding used for vertical displacement measurements.

Measurements of vibration parameters in terms of displacements, velocities, and accelerations in the vertical direction were performed on modified concrete sleepers with USPs in an intact area. Two measuring points in an intact/undamaged state at the distance of 5 m were chosen, one in the area where the rail was not welded (MP1) and the other directly at the location of the weld (MP2). In both cases, inductive displacement sensors were used to measure vertical displacements using a stationary reference structure in the form of cantilevered scaffolding (Figure 2), placed about 3 m from the tracks in an area where the ground vibration amplitudes due to the passing of the train were negligible.

Additionally, to identify the differences between the responses of standard concrete and wooden sleepers, synchronous measurements on two adjacent standard concrete and wooden sleepers were performed at measuring point MP3. These measurements were conducted in a partially damaged area where the phenomenon of floating sleepers had begun to emerge. As is well known, ballast fouling, which often accompanies this phenomenon, significantly reduces track serviceability and poses a risk to the safe operation of trains. The final stage of the phenomenon occurs when the sleepers lose contact with the ground, leaving them practically hanging on the rails [14,15,16].

### 2.2. Equipment for Vibration Measurements

For measurements of vibration parameters, Hottinger Baldwin Messtechnik, Darmstadt, Germany (HBM) inductive sensors of type WA-50 mm and measuring amplifiers from the same manufacturer, HBM MGCplus and HBM QuantumX MX840A, were used. Catman AP software Ver. 4.2.2, developed for HBM measurement amplifiers, was used to capture and and Catman AP software Ver. 5.6.1.12 was used for final analysis of the measurement results. From the measured signals of the vertical displacements of the sleepers, velocities and accelerations were evaluated by numerical differentiation. The sampling frequency for all measurements was 600 Hz. Acceleration was initially measured using accelerometers; however, excessive amplitudes caused by steel-on-steel impacts during train passages frequently exceeded the sensor limits. To avoid saturation, inductive displacement sensors were employed, despite their lower accuracy and limited high-frequency response. Given that the study’s early focus was on floating sleeper phenomena and track settlement, displacement measurements were sufficient. Moreover, displacement data offer a practical advantage, as numerical differentiation is generally more stable and straightforward than integrating acceleration signals.

## 3. Results

### 3.1. Influence of Welds on the Response of Modified Concrete Sleepers in an Undamaged Area

In the track line Ljubljana–Vrhnika on the section by Borovnica, measurement sites were chosen in such a way that potential differences in measured vibration parameters due to the presence of welds in the rail could be evaluated in an undamaged state. Measurements were performed on modified concrete sleepers with USPs. Specifications of USPs are listed in Table 1.

**Figure 3:**
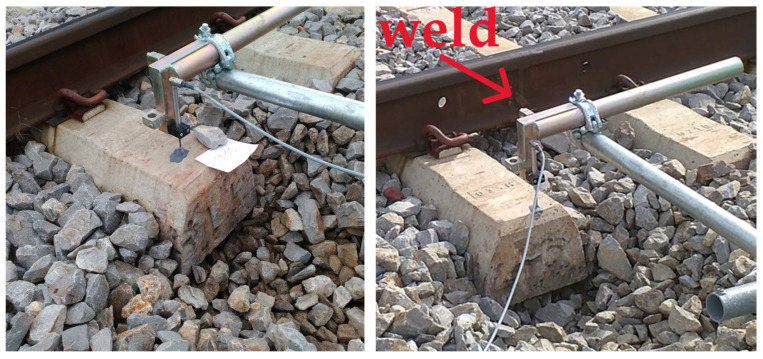
Measuring points MP1 ((**left**), no weld on the rail) and MP2 ((**right**), weld on the rail) on concrete sleepers with USPs in the undamaged area.

Both measuring points, MP1 and MP2, were selected in an undamaged, intact area of the track at the distance of 5 m. The main difference between the two measuring points was that at measuring point MP1, there was no weld, and at MP2, weld was present on the rail (Figure 3).

**Figure 4:**
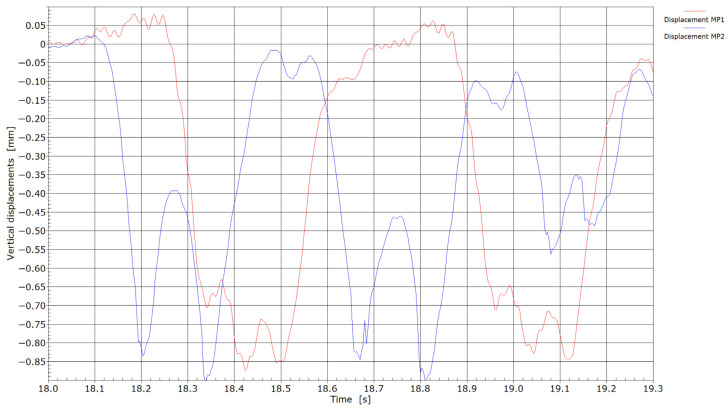
Displacements by the passage of locomotives through measuring points MP1 and MP2.

Displacements measured by the passing of two different locomotives over measuring points MP1 and MP2 are shown in Figure 4. No direct speed measurements were taken; however, trains operating on these sections typically travel at approximately 60 km/h. At measurement point MP1, the response of a six-axle locomotive 363 with a total mass of 114 tons was recorded. At measurement point MP2, measurements were conducted for a Siemens Taurus four-axle locomotive with a mass of 80 tons. Despite differences in locomotive type and axle configuration, the axle loads were similar: approximately 19 tons at MP1 and 20 tons at MP2. The measured displacements at both locations remained within a comparable range, with maximum values reaching approximately 0.9 mm.

**Figure 5:**
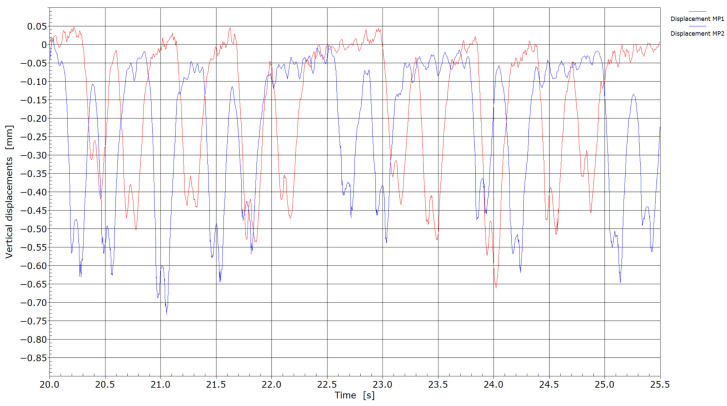
Displacements by the passage of trains through measuring points MP1 and MP2.

The recordings presented in Figure 5 correspond to freight wagons pulled by locomotive 363 at measuring point MP1 and freight wagons pulled by the Siemens Taurus locomotive at measuring point MP2. In both cases, the locomotives were hauling freight wagons loaded with ore. As shown in Figure 5, no significant differences in the measured displacements were observed during the passage of the wagons; however, as expected, the displacement values were lower compared to those recorded during the passage of the locomotives.

**Figure 6:**
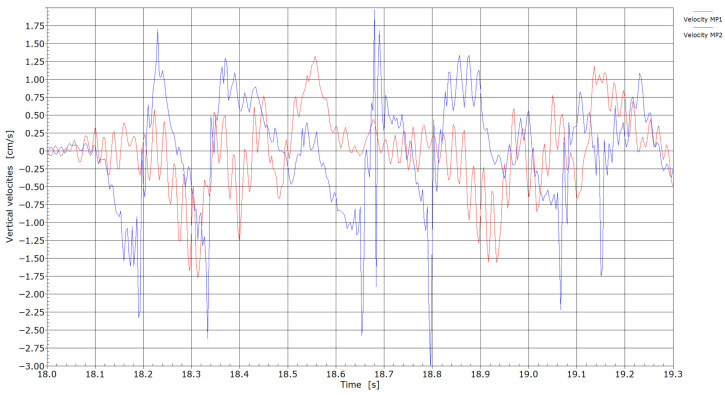
Velocities by the passage of locomotives through measuring points MP1 and MP2.

**Figure 7:**
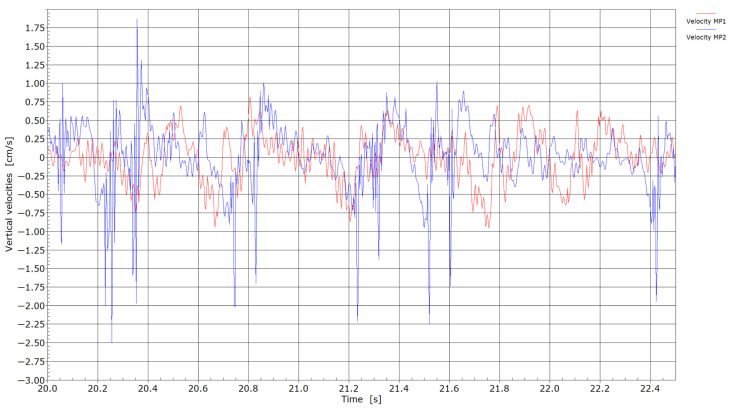
Velocities with the passage of trains through measuring points MP1 and MP2.

**Figure 8:**
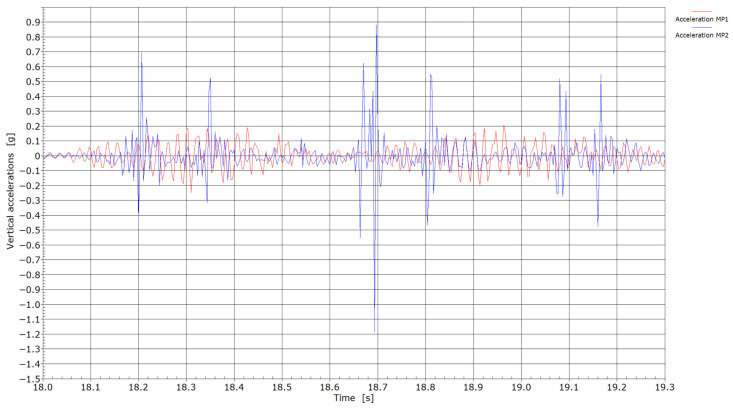
Accelerations with the passage of locomotives through measuring points MP1 and MP2.

**Figure 9:**
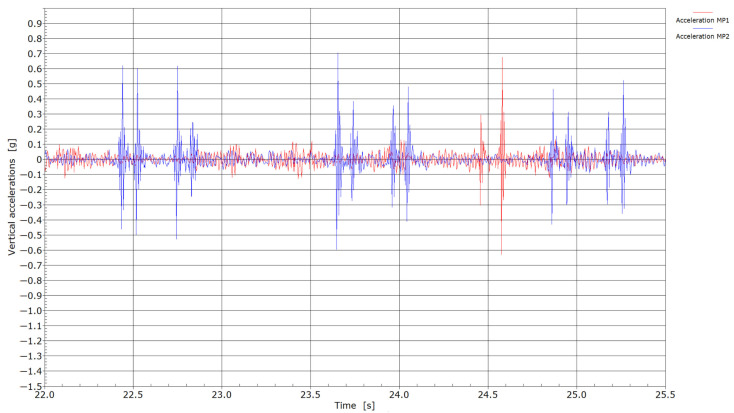
Accelerations by the passage of trains through measuring points MP1 and MP2.

Figure 6, Figure 7, Figure 8 and Figure 9 present velocity and acceleration data. In both cases, the differences between the two measurement points become more pronounced. Given that the axle loads of locomotives hauling freight wagons are similar and their travel speed is limited to 60 km/h, these variations in velocity and acceleration responses can be attributed to differences due to welding conditions. The pronounced acceleration peaks observed in Figure 8 and Figure 9 further suggest the presence of flat wheel damage. During the passing of locomotives, the maximum absolute velocities at measuring point MP2 were in the range of 1.75 and 3.0 cm/s, and between 1.25 and 1.75 cm/s at measuring point MP1. With the passing of trains, maximum absolute velocities at measuring point MP2 reached 2.0 cm/s or more; meanwhile, at measuring point MP1, absolute peaks were in the range of 0.6 to 0.9 cm/s. It is evident that significantly lower velocities were obtained at measuring point MP1 (without weld) compared to measuring point MP2 (presence of weld).

From the accelerations measured during the passing of locomotives (Figure 8), a much more continuous response without pronounced peaks with values in the range of ±0.2 g was recorded at measuring point MP1 compared to measuring point MP2, where individual peak values reached up to ±0.5 g. The highest recorded absolute value of 1.2 g could be the consequence of a flat wheel on one of the axles of the locomotive. Also, with passing trains (Figure 9), considerably higher accelerations in general were measured at measuring point MP2 compared to MP1. With the passage of trains, two major peaks (at 24.45 and 24.55 s) were recorded when passing through MP1, indicating possible flat wheel damage on these two axles.

As can be seen from the obtained results, the influence of welds was not recorded by measurements of displacements, but it is obvious from the recorded velocities, and even more pronounced with the measured accelerations. The degradation of ballast track and concrete sleepers equipped with USPs near welds progressed more slowly and to a lesser extent during the initial stages of track operation compared to conventional concrete sleepers. Field data from long-term monitoring indicate that USPs reduce ballast deterioration and extend tamping intervals, thereby lowering life-cycle maintenance requirements and yielding an approximate 40% increase in service life when a 30 cm thick ballast bed is used [17]. However, over prolonged operational periods and with the accumulation of material fatigue, the protective benefits of USPs diminish, and degradation ultimately occurs, particularly in heavily loaded track sections [12,13].

### 3.2. Differences Between the Responses of Standard Concrete and Wooden Sleepers in the Partly Damaged Area

**Figure 10:**
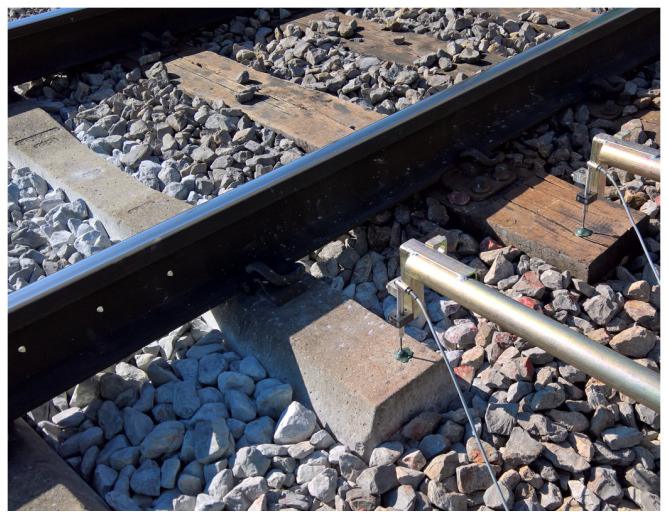
Measuring point MP3 on concrete and wooden sleeper in partly damaged area.

The reconstruction of damaged parts of railway tracks in Slovenia usually involves the replacement of worn concrete sleepers with new wooden ones, which are known to have lower stiffness compared to concrete sleepers and enable the imprinting of ballast (Figure 1). At measuring point MP3, the synchronous measurements on two adjacent standard concrete and wooden sleepers were performed in a partly already damaged state. Measurements were performed on a standard concrete sleeper at a distance of 1.50 m from the weld and on the wooden sleeper at 2.10 m from the weld. Figure 10 shows the setup of measurements and visible damage to the ballast bed. The measurement area represents the transition from the wooden to the concrete sleeper area, where the phenomenon of floating sleepers is beginning to appear.

**Figure 11:**
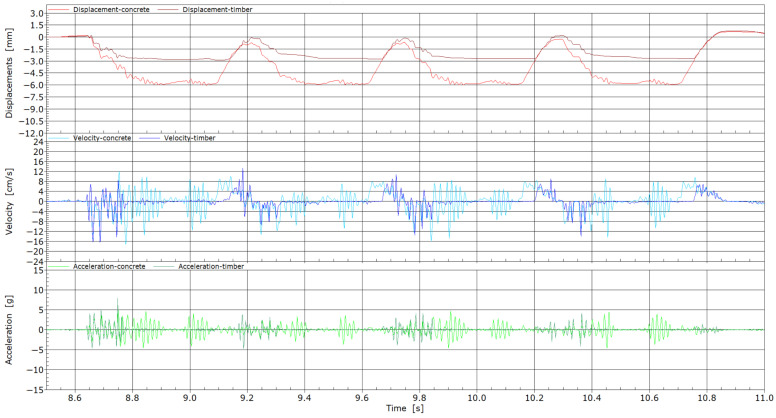
Displacements, velocities, and accelerations from the passage of two locomotives through measuring point MP3.

In Figure 11, the comparison of vibration parameters at measuring point MP3 when two connected four-axle locomotives passed over two adjacent sleepers, concrete and wooden, is shown. The response of concrete and wooden sleepers differed, especially in the case of displacements. The concrete sleeper showed increased values of displacements visible upon the passage of each of the axles. It was also emphasized that high-frequency vibrations in the sleeper upon each axle’s arrival at the sleeper location can be seen. The maximum displacement value of the concrete sleeper was 6 mm. For the wooden sleeper, a more continuous response was obtained; there were fewer high-frequency vibrations, and the displacements were more than 50% smaller in comparison with those of the concrete sleeper. The sleepers’ material differences can amplify settlements and support inconsistencies in areas prone to the floating sleeper phenomenon. Concrete sleepers, being much stiffer and heavier than wooden ones, tend to cause uneven load distribution, especially in transition zones. Additionally, the interaction between the sleeper and the ballast is influenced by the material properties of the sleeper. However, the primary cause of displacement differences can be attributed to the initiation of the floating sleeper phenomenon near the weld location. In contrast, the more compliant behavior of wooden sleepers has been shown to mitigate this effect, providing a more uniform load transfer and thereby reducing the risk of further ballast deterioration. No significant differences were observed between the two types of sleepers in terms of measured velocity and acceleration. Measured velocities range between 12 and −16 cm/s, and accelerations between 5 and −5 g.

**Figure 12:**
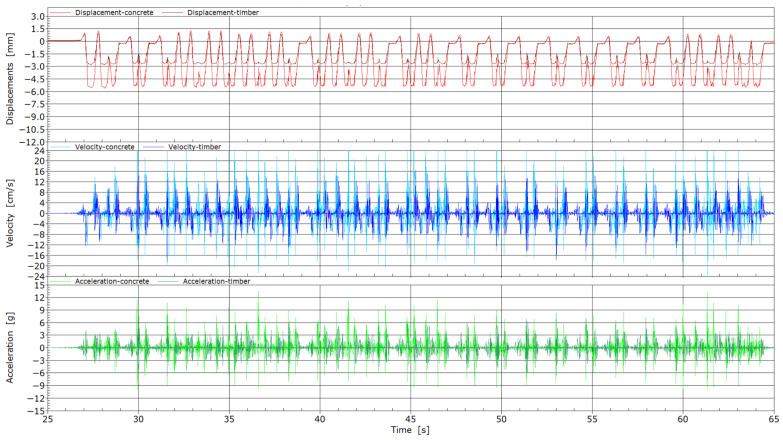
Displacements, velocities, and accelerations from the passage of train composition through measuring point MP3.

Measurements of vibration properties at measurement point MP3 were also carried out with the passing of train composition, and are presented in Figure 12. Even in this case, the differences between two types of sleepers were evident in the measurements of displacements, which were considerably smaller with the wooden sleeper compared to the concrete one. For the wooden sleeper, a displacement of −2.8 mm was reached, while with the concrete sleeper the value was twice as high and amounted to −5.5 mm. Displacements in the case of the wooden sleeper were also more continuous and smoother, without high-frequency vibrations, as in case of the concrete sleeper. Additionally, for the wooden sleeper, the shape of the displacement graph does not reveal the transition of individual axes, as in the case of the concrete sleeper. From the measurements of velocities, higher values were achieved by concrete sleepers, with values ranging between 20 and 24 cm/s, while for the wooden sleepers, maximum absolute values between 12 and 16 cm/s were achieved. Accelerations for the wooden sleepers mostly ranged from 0 to 3 g, and reached 6 g in some parts. For the concrete sleeper, absolute values from 9 to 12 g were reached.

From the passage of two locomotives (Figure 11), the differences between the two types of sleepers are visible, mainly in the measurements of displacements, but there are no significant differences in measured velocities and accelerations. The passage of the train composition (Figure 12) reveals distinct differences between wooden and concrete sleepers, as evidenced by the recorded vibration responses in terms of displacement, velocity, and acceleration. These parameters—displacement, velocity, and acceleration—are significantly higher in the case of concrete sleepers compared to wooden ones. Specifically, the velocity of concrete sleepers reaches up to 24 cm/s, whereas that of wooden sleepers generally does not exceed 14 cm/s over a broader area. Similarly, the acceleration of concrete sleepers peaks at 14 g, while for wooden sleepers it reaches only up to 7 g. These results suggest that wooden sleepers provide a better mitigation of ballast bed damage in critical sections compared to their concrete counterparts. This finding also aligns with current railway maintenance practices in Slovenia, where concrete sleepers in critical zones are replaced with wooden ones following the onset and progression of ballast degradation. The railway section toward Luka Koper in Divača, where data were collected at the MP3 measuring point, experiences high traffic volumes. Due to the presence of multiple critical points, regular maintenance is required, leading to weekly track closures. During these interventions, concrete sleepers are systematically replaced with wooden alternatives, which have proven more effective at distributing contact pressures. Additionally, the degradation of the track bed is significantly influenced by the substandard quality of the ballast material. To assess potential differences in the sub-sleeper layers, Ground Penetrating Radar (GPR) measurements were conducted at the MP3 site [1], revealing no significant differences in the subsurface layers beneath the concrete and wooden sleepers.

## 4. Discussion and Possible Mitigation Measures

As shown by the results of several investigations, the installation of an elastomeric material between the sleeper and the ballast bed, such as a rubber layer, can improve the performance of the concrete sleepers. This option helps in reducing the crushing of ballast stones and increases the resistance against transverse movements [9,10,11]. However, modified concrete sleepers with USPs do not entirely solve the problem in areas where welds are present in the rail. This was shown by measurements of the response of concrete sleepers with USPs at an undamaged area (MP1 and MP2), as presented in Section 3. At the weld (MP2), the velocities were higher by a factor of 1.7, and the accelerations even by a factor of 2.5 compared to the area without a weld (MP1). The probability of damage formation in the welded area is, therefore, despite the use of modified concrete sleepers, higher than in remaining areas. In undamaged areas, the lower stiffness of the wooden sleepers, combined with the tendency for ballast particles to embed into the sleeper surface, may lead to greater displacements compared to concrete sleepers. However, as demonstrated by synchronous measurements conducted on standard concrete and wooden sleepers in a partially damaged section (MP3), these same material properties exhibit a beneficial effect: they help mitigate damage and result in reduced displacements relative to concrete sleepers.

Considering the results obtained from the field measurements, the track’s response in the weld area is stiffer, which leads to higher velocities and accelerations. Although additional research and analysis are required to solve this problem, it can be concluded that the solution should aim to reduce vibration parameters to minimize the possibility of damage formation. One possibility is modifying the ballast track, or its separate parts, in the limited area where damage is expected. The sleepers should be flexible enough to dampen dynamic loads and have a larger contact surface to prevent or limit the crushing of ballast stones in contact with the sleeper. This could be achieved by increasing the contact surface and/or enabling the ballast stones to imprint into the contact surface. In this regard, the sleeper could be made of two layers: a softer material on the bottom and a harder material on the top. Given the favorable response obtained in measurements of wooden sleepers, another possibility would be to use sleepers made of higher-quality wood with substantial yet environmentally friendly impregnation, which would extend their lifespan. One promising option for improving the ballast layer’s response is installing an elastomeric layer into the track structure below the ballast bed. There are reports of the beneficial effects of under-ballast mats, primarily in reducing the impact of vibrations and damage to the ballast track, which has resulted in reduced maintenance costs [18]. A recent study showed that adding over 10% rubber granules from waste tires to ballast improves rail track performance by reducing dilation, degradation, and breakage. Rubber particles sized 9.5–19 mm with similar angularity can protect aggregates and control fouling. However, excess rubber leads to larger initial settlements due to particle compression [19]. As part of further research, instead of wood, which has limited durability, metal foam could be used at the contact location between the sleeper and the ballast in critical areas. Metal foam has been gaining ground in the automotive industry for some time now, because it is extremely light and a very good energy dissipator, and it is also increasingly cheaper. It yields under point loads and withstands high pressures over a large area [20,21], similar to wood. Of course, it is still too expensive to use it as a base for sleepers on entire railway lines, but sleepers with a metal foam underpad in the weld area could function similarly to wooden ones. At the same time, they could represent a more durable alternative.

In addition to implementing appropriate mitigation measures, it would be advisable to regularly monitor potentially critical areas. This approach, which identifies problematic locations and implements appropriate mitigation measures before damage occurs, improves the management of railway infrastructure. This would improve railway safety and reduce maintenance costs. At the same time, it would be necessary to test the proposed improvements in a laboratory setting first. If those tests are successful, test fields should be set up on critical sections of railway lines to evaluate the effectiveness of the proposed solutions in practice.

## Figures and Tables

**Table 1:** Specifications of the USPs used at measuring points MP1 and MP2.

Specifications of USPs	
Producer:	Pandrol
Material:	Resin-bonded rubber (recycled)
Geometry:	Flat
Type:	Soft pad
Thickness:	10 mm
Density:	1025 kg/m^3^
Weight:	±1.5 kg/m^2^
Static bedding modulus C_stat_:	0.13 N/mm^3^ (typical axle load 225 kN)
Bond strength:	>0.5 MPa

## Data Availability

Data supporting reported results can be found at https://informativni.fgpa.um.si/fakulteta/oddelki-in-katedre/katedra-za-gradbeno-mehaniko/(accessed on 25 June 2025).

## Abbreviations

The following abbreviations are used in this manuscript:

HBM	Hottinger Baldwin Messtechnik
USPs	Under-sleeper pads
